# The Application of NCaRBS to the Trendelenburg Test and Total Hip Arthroplasty Outcome

**DOI:** 10.1007/s10439-014-1231-1

**Published:** 2015-01-23

**Authors:** Gemma Marie Whatling, C. A. Holt, M. J. Beynon

**Affiliations:** 1 Cardiff School of Engineering, Cardiff University, Queen’s Buildings, The Parade, Cardiff, CF24 3AA UK; 2 Cardiff Business School, Cardiff University, Cardiff, UK

**Keywords:** *N*-state classification, Dempster–Shafer theory (DST) of evidence, Ignorance, Pignistic probability, Total hip arthroplasty

## Abstract

This paper compares the frontal plane hip function of subject’s known to have had hip arthroplasty *via* either the lateral (LA) or posterior (PA) surgical approaches and a group of subjects associated with no pathology (NP). This is investigated through the Trendelenburg test using 3D motion analysis and classification. Here, a recent development on the Classification and Ranking Belief Simplex (CaRBS) technique, able to undertake *n*-state classification, so termed NCaRBS is employed. The relationship between post-operative hip function measured during a Trendelenburg Test using three patient characteristics (pelvic obliquity, frontal plane hip moment and frontal plane hip power) of LA, PA and NP subjects are modelled together. Using these characteristics, the classification accuracy was 93.75% for NP, 57.14% for LA, 38.46% for PA. There was a clear distinction between NP and post-surgical function. 3/6 LA subjects and 6/8 PA subjects were misclassified as having NP function, implying that greater function is restored following the PA to surgery. NCaRBS achieved a higher accuracy (65.116%) than through a linear discriminant analysis (48.837%). A Neural Network with two-nodes achieved the same accuracy (65.116%) and as expected was further improved with three-nodes (69.767%). A valuable benefit to the employment of the NCaRBS technique is the graphical exposition of the contribution of patient characteristics to the classification analysis.

## Introduction

The considered problem throughout this paper is total hip arthroplasty (THA), and is concerned with identifying differences in post-operative biomechanical function following two common surgical approaches to THA; namely the McFarland–Osborne—direct Lateral Approach (LA)^23^ and the Moore—southern exposure posterior approach (PA).^24^ During both these procedures the hip joint is replaced with prosthetic components; however the joint can be accessed from different directions. Each surgical approach to THA compromises different muscles and passive constraints surrounding the hip joint. It is important to provide biomechanical feedback *in vivo* on different surgical approaches and identify any changes in frontal plane function associated with them.^18^,^32^

This paper also exposits a development on the binary ‘2-state’ classification, classification and ranking belief simplex (CaRBS),^4^,^5^,^7^ which is able to undertake *n*-state classification, termed *n*-state classification and ranking belief simplex (NCaRBS). *N*-state classification is very desirable in biomechanics research where two or more conditions or treatment groups are being considered. CaRBS and NCaRBS, whose operations are based on the Dempster–Shafer theory of evidence,^10^,^29^ offer an approach to object classification modelling based on uncertain or evidential reasoning.^9^,^26^ Early applications of the CaRBS technique include corporate failure and bank rating prediction,^4^,^5^ osteoarthritic knee analysis,^7^,^19^ THA outcome from gait,^32^ bird gender classification^6^ and student performance analysis.^20^

The development of NCaRBS from CaRBS, is an important step, with its exposition describing a much more general classification technique than previously existing with CaRBS. As the technical details will show, the progression from CaRBS to NCaRBS is homogeneous, with the same process for the combination of evidence (using Dempster’s combination rule) used throughout a NCaRBS analysis. An important continuation from CaRBS, is that with NCaRBS there remains the clear opportunity to visualise the contribution of characteristics, in a variety of ways, throughout a NCaRBS analysis. This is extremely useful when assessing biomechanical measures, as it provides insight into how patient characteristics contribute to an overall classification of function for each treatment group.

With respect to THA, NCaRBS is used here to characterise biomechanical function from a one leg stance, commonly termed the Trendelenburg test,^18^ to discern between subjects associated with the defined surgery types (the considered states in terms of NCaRBS), LA, PA and those with no-pathology (NP) (to simplify writing style NP is here considered a surgery type). The simultaneous analysis of these three surgery types provides effective feedback on NP and post-operative hip function, both in terms of discerning between the surgical approaches LA and PA, and each of them against NP.

This paper describes the THA problem, the NCaRBS technique and its application in relation to the THA problem, including emphasis on the elucidation of the contribution of Trendelenburg stance characteristics in the describing evidence they offer in classifying subjects to the three surgery types.

To offer a level of comparison with the NCaRBS results, two techniques previously employed in studies involving biomechanical data were also considered, namely linear discriminant analysis (LDA)^21^,^28^ and neural networks (NNs).^1^,^3^,^16^ Comparisons were made with respect to the level of correct classification when using LDA, and level of correct classification and fit in the case of NNs.

### Total Hip Arthroplasty and Trendelenburg Test

THA is a procedure to remove the diseased parts of an osteoarthritic hip joint and replace them with prosthetic articulating components. The hip joint is commonly accessed through incisions either lateral or posterior to the joint. The primary cause of gait disturbances following THA is the disruption of the abductor structures. The abductors play a crucial role during the single stance phase in gait by controlling hip abduction and pelvic obliquity. Each surgical approach, lateral (LA) or posterior (PA), compromises different structures around the hip joint, affecting frontal plane function to different extents.

With respect to PA, the abductor mechanism is preserved and the posterior joint capsule and external rotator muscle group are compromised, affecting posterior and lateral stability. In contrast, the LA preserves the posterior capsule and part of the insertion of the gluteus medius into the greater trochanter. If migration of the abductor tendon occurs during healing, this introduces a change in the mechanical ability of the abductors affecting frontal plane function. Compromising the abductor muscle group can diminish the mechanical ability of the abductor mechanism in controlling the hip joint function and pelvis orientation in the frontal plane.^2^,^12^ In a study of abductor strength, the PA was found to lead to more normal hip abductor muscle strength than following an anterolateral approach.^17^ There is currently no consensus on the approach to use to provide optimum patient outcome and thus THA outcome warrants further investigation.

The Trendelenburg test,^18^ which is a standard clinical assessment to determine the integrity of hip abductor function, is an examination of a subject’s posture whilst they stand on one leg. The action of changing from a two-leg to a single-leg stance shifts the line of gravity of the superincumbent body, producing moments about the hip that must be balanced by a moment arising from the force of the abductor muscles. If the pelvis on the unsupported side is raised then the Trendelenburg test is negative. In the case of a positive Trendelenburg test, the pelvis on the unsupported side falls below the horizontal position indicating abductor weakness. This action moves the line of gravity towards the supporting hip, reducing the moment lever arm and consequently the moment that must be counteracted by the abductors and other passive structures to maintain stability. Therefore three measureable characteristics are useful to quantify during this test, pelvic obliquity which is the angle of the pelvis in the frontal plane, fontal plane hip moment and frontal plane hip power.

The Trendelenburg test is used routinely in a clinic to assess hip strength and stability and is the focus of this study. The clinician scores this test based on the patient’s ability to perform the test and retain pelvic elevation over time. Baker and Bitounis,^2^ using a Trendelenburg test to assess abductor strength, reported abductor weakness following the LA indicated by a higher incidence of positive Trendelenburg tests^18^ (where the unsupported pelvis either remained horizontal or dropped below the horizontal position) as compared to following the PA, whereas Downing *et al*.,^13^ in comparing the LA and PA, did not find significant differences in abductor strength. Both studies used electromyography for their investigation. In a study using three dimensional (3D) motion analyses of hip function during a Trendelenburg test,^32^ hip power and moment was on average greatest for NP followed by PA and then LA, with statistical differences found between the NP and PA cohorts. No significant differences in pelvis elevation were found. This study further investigates whether functional differences can be detected during a Trendelenburg test that are associated with LA, PA and NP, using 3D motion analysis data from Whatling *et al.* 32 and the NCaRBS technique. This is the first time that the LA, PA and NP cohorts have been classified using NCaRBS and data from the clinical Trendelenburg test only.

### Background to the NCaRBS Technique

The NCaRBS, is a non-parametric evidence-based technique, able to classify objects to a number of states (rather than just two states when using CaRBS^4^,^5^,^7^), using the evidence from a series of characteristics describing them. The description of this development (NCaRBS) will follow the description of the CaRBS technique exposited in previous papers by Beynon.^4^,^5^,^7^ Referral to these studies will aid the reader in the understanding of the role played by the Dempster–Shafer theory (DST) of evidence,^10^,^29^ a nascent methodology, underpinning CaRBS (and NCaRBS), which has its own unique formulisation. With DST, the basis of its operations is formulated around the formation of bodies of evidence (BOEs), made up of mass values representing the levels of exact belief (mass values) in associated focal elements. Their (BOE) construction, subsequent combination and representation are at the heart of the operations in the NCaRBS, technique.

**Figure 1:**
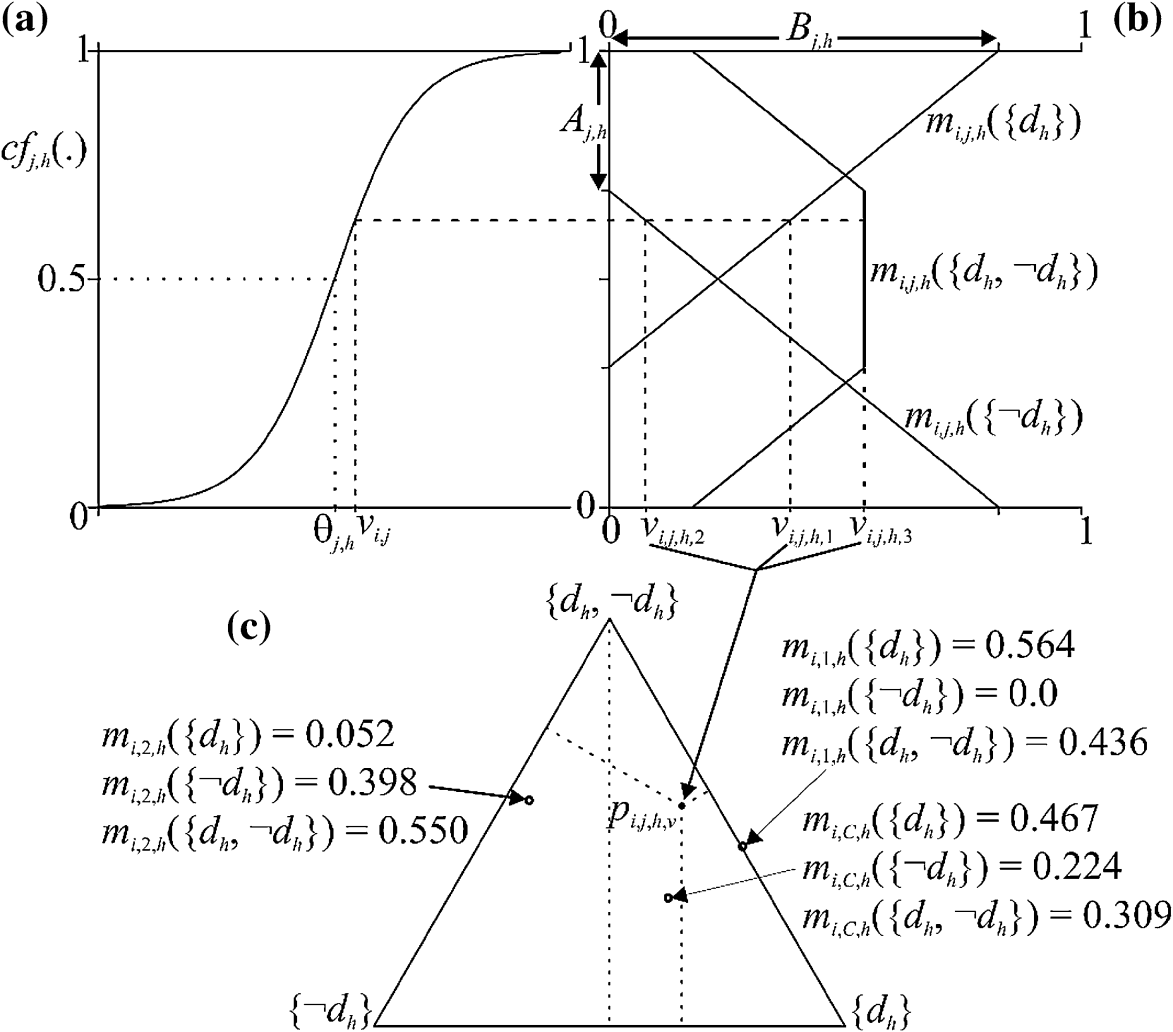
Stages within the NCaRBS technique for the construction and representation of a constituent BOE *m*_*i*,*j*,*h*_(·) (Concerned with *i*th object, *j*th characteristic, *h*th state), from characteristic value *v*_*i*,*j*_

The general remit of the NCaRBS development of CaRBS is to model the association of *n*_O_ objects $(o_{ 1} ,o_{ 2} , \ldots ,o_{{n_{\text{O}} }} )$, to *n*_D_ states $(d_{ 1} ,d_{ 2} , \ldots ,d_{{n_{\text{D}} }} )$, based on their description by *n*_C_ characteristics $(c_{ 1} ,c_{ 2} , \ldots ,c_{{n_{\text{C}} }} )$. With respect to DST, throughout the analysis the considered frame of discernment (FoD) Θ is the set of *n*_D_ states, namely $\varTheta = \, \{ d_{ 1} ,d_{ 2} , \ldots ,d_{{n_{\text{D}} }} \}$. The characteristic-based evidence in the classification process is expressed through the construction of constituent BOEs from an object’s characteristic values *v*_*i*,*j*_ (*i*th object, *j*th characteristic), to discern between the object’s association to a state (say {*d*_*h*_}), its complement $({\text{not}}\;d_{h} - \, \left\{ {\neg d_{h} } \right\} \equiv \{ d_{ 1} ,d_{ 2} , \ldots ,d_{h - 1} ,d_{h + 1} , \ldots ,d_{{n_{\text{D}} }} \} )$ and a level of concomitant ignorance ($\left\{ {d_{h} , \, \neg d_{h} } \right\} \equiv \varTheta$—evidence not able to be discerned between *d*_*h*_ and ¬*d*_*h*_). The construction of a constituent BOE, defined *m*_*i*,*j*,*h*_(·) (*i*th object, *j*th characteristic, *h*th state), discerning between {*d*_*h*_}, {¬*d*_*h*_} and {*d*_*h*_, ¬*d*_*h*_}, can be best described by reference to Fig. 1.

In Fig. 1, stage (a) shows the transformation of a characteristic value *v*_*i*,*j*_ into a confidence value *cf*_*j*,*h*_(*v*_*i*,*j*_) over the range 0–1, here using a sigmoid function, *cf*_*j*,*h*_(*v*_*i*,*j*_) = 1/(1 + exp(−*k*_*j*,*h*_(*v*_*i*,*j*_ − *θ*_*j*,*h*_)), with control parameters *k*_*j*,*h*_ and *θ*_*j*,*h*_. Stage (b) transforms a *cf*_*j*,*h*_(*v*_*i*,*j*_) into a *constituent* BOE *m*_*i*,*j*,*h*_(·), made up of the three mass values, *m*_*i*,*j*,*h*_({*d*_*h*_}), *m*_*i*,*j*,*h*_({¬*d*_*h*_}) and *m*_*i*,*j*,*h*_({*d*_*h*_, *¬d*_*h*_}), defined by (see Beynon^5^ and Safranek *et al.* 27):

$$m_{i,j,h} \left( {\{ d_{h} \} } \right) = \hbox{max} \left( {0,\frac{{B_{j,h} }}{{1 - A_{j,h} }}cf_{j,h} \left( {v_{i,j} } \right) - \frac{{A_{j,h} B_{j,h} }}{{1 - A_{j,h} }}} \right),$$

$$m_{i,j,h} \left( {\{ \neg d_{h} \} } \right) = \hbox{max} \left( {0,\frac{{ - B_{j,h} }}{{1 - A_{j,h} }}cf_{j,h} \left( {v_{i,j} } \right) + B_{j,h} } \right),$$

$${\text{and }}m_{i,j,h} \left( {\{ d_{h} ,\neg d_{h} \} } \right) = 1 { } - m_{i,j,h} \left( {\{ d_{h} \} } \right) \, - m_{i,j,h} \left( {\{ \neg d_{h} \} } \right),$$

where *A*_*j*,*h*_ and *B*_*j*,*h*_ are two further control parameters. Stage (c) shows a BOE *m*_*i*,*j*,*h*_(·); *m*_*i*,*j*,*h*_({*d*_*h*_}) = *v*_*i*,*j*,*h*,1_, *m*_*i*,*j*,*h*_({¬*d*_*h*_}) = *v*_*i*,*j*,*h*,2_ and *m*_*i*,*j*,*h*_({*d*_*h*_, *¬d*_*h*_}) = *v*_*i*,*j*,*h*,3_, can be represented as a simplex coordinate (*p*_*i*,*j*,*h*,*v*_) in a simplex plot (equilateral triangle). For a simplex plot with unit side, with vertices (0, 0), (1, 0) and ($0. 5, \, 0. 5 { }\sqrt 3$), the *p*_*j*,*i*,*v*_ simplex coordinate (*x*_p_, *y*_p_) is given by *x*_p_ = *v*_*j*,*i*,1_ + 0.5*v*_*j*,*i*,3_ and $y_{\text{p}} = 0.5\,\sqrt 3 \;v_{j,i,3.}$.

Based on the produced constituent BOEs, *m*_*i*,*j*,*h*_(·) *j* = 1,…,*n*_C_ and *h* = 1,…,*n*_D_, describing the evidence from the individual object *o*_*i*_ characteristics, Dempster’s rule of combination is used to combine them to allow a final association of each object to each of the *n*_D_ states. There are a number of ways of combining constituent BOEs within NCaRBS (offering different inference to the evidence from the characteristics they are combining). In general, the combination of two constituent BOEs, $m_{{i,j_{1} ,h}} ( \cdot )$ and $m_{{i,j_{2} ,h}} ( \cdot )$, for the same object (*o*_*i*_), from two characteristics (*c*_*j*,1_ and *c*_*j*,2_) and single state (*d*_*h*_), is defined $(m_{{i,j_{1} ,h}} \oplus m_{{i,j_{2} ,h}} )( \cdot )$, and results in a combined BOE whose three mass values (and focal elements) are given by (see Dempster^10^; Shafer^29^):

$$(m_{{i,j_{1} ,h}} \oplus m_{{i,j_{2} ,h}} )( \cdot ) = \left\{ {\begin{array}{ll} 0 & {x = \emptyset } \\ {\frac{{\sum\limits_{{s_{1} \cap s_{2} = x}} {m_{{i,j_{1} ,h}} \left( {s_{1} } \right)m_{{i,j_{2} ,h}} \left( {s_{2} } \right)} }}{{1 - \sum\limits_{{s_{1} \cap s_{2} = \emptyset }} {m_{{i,j_{1} ,h}} \left( {s_{1} } \right)m_{{i,j_{2} ,h}} \left( {s_{2} } \right)} }}} & {x = \emptyset } \\ \end{array} } \right.$$

where *s*_1_ and *s*_2_ are focal elements from the constituent BOEs, $m_{{i,j_{1} ,h}} ( \cdot )$ and $m_{{i,j_{2} ,h}} ( \cdot )$, respectively. An example of the combination of the BOEs, *m*_*i*,1,*h*_(·) and *m*_*i*,2,*h*_(·), and their combination to the combined BOE *m*_*i*,C,*h*_(·) is illustrated graphically based on their simplex coordinate positions in the simplex plot in Fig. 1c.

This combination process can then be performed iteratively to combine all the characteristic-based evidence, namely the constituent BOEs *m*_*i*,*j*,*h*_(·) *j* = 1,…,*n*_C_, describing an object *o*_*i*_ to a single state *d*_*h*_, producing a *state* BOE, defined *m*_*i*,−,*h*_(·) (the ‘dashed line’ subscript shows the index not kept constant during the combination of the respective constituent BOEs). Alternatively, through the combination of the constituent BOEs *m*_*i*,*j*,*h*_(·) *h* = 1,…,*n*_D_, for a single object *o*_*i*_ and characteristic *c*_*j*_, the discernment over the different states produces a *characteristic* BOE, defined *m*_*i,j,*−_(·).

The respective state BOEs (or characteristic BOEs) can be combined in a similar way to the combination of the constituent BOEs to bring together the relevant evidence. Moreover, for each object *o*_*i*_, an *object* BOE is evaluated, defined *m*_*i*,−,−_(·) (reduced to *m*_*i*_(·) for brevity), by either combining the state BOEs *m*_*i*,−,*h*_(·) *h* = 1,…,*n*_D_, or the characteristic BOEs *m*_*i,j,*−_(·) *j* = 1,…,*n*_C_, again using Dempster’s rule of combination. It is the object BOEs *m*_*i*_(·) *i* = 1,…,*n*_O_, that contain the evidence towards the predicted associations of the *n*_O_ objects to the *n*_D_ states.

The object BOEs (and other BOEs) are made up of mass values associated with focal elements, which are the power set of the ${\text{FoD}}\;\varTheta ( = \, \{ d_{ 1} ,d_{ 2} , \ldots ,d_{{n_{\text{D}} }} \} )$ minus the empty set. As such, the evidence in an object BOE cannot directly collate the associations of an object to the individual *n*_D_ states considered. To construct the values depicting the levels of association of objects to the individual states $d_{ 1} ,d_{ 2} , \ldots ,d_{{n_{\text{D}} }}$, the pignistic probability function—*BetP*_*i*_(·) is utilised.^11^ For an object *o*_*i*_, using the object BOE *m*_*i*_(·), the $BetP_{i} \left( {d_{h} } \right) = \sum\limits_{{\mathop {s_{1} \subseteq \{ d_{1} ,d_{2} , \ldots \} }\limits_{{s_{j} \cap \{ d_{k} \} \ne \emptyset }} }} {m_{i} \left( {s_{j} } \right)/\left| {s_{j} } \right|}$ value represents the level of pignistic probability associating that object with the state *d*_*h*_. It follows, the series of pignistic probability values $BetP_{i} \left( {d_{ 1} } \right),BetP_{i} \left( {d_{ 2} } \right), \ldots ,BetP_{i} (d_{{n_{\text{D}} }} )$, dictates the predicted associations of the object *o*_*i*_ to the considered states $d_{ 1} ,d_{ 2} , \ldots ,d_{{n_{\text{D}} }}$, respectively.

The effectiveness of the NCaRBS technique, in terms of modelling objects’ known associations to a series of states, based on their associated characteristics, is governed by the values assigned to the incumbent control parameters *k*_*j*,*h*_, *θ*_*j*,*h*_, *A*_*j*,*h*_ and *B*_*j*,*h*_, *j* = 1,…,*n*_C_ and *h* = 1,…,*n*_D_ (see Fig. 1 for a description of their roles). This necessary configuration, that is, assignment of values to the control parameters, is considered as a constrained optimisation problem, and solved here using Trigonometric Differential Evolution (TDE).^14^

In summary, TDE is an evolutionary algorithm that iteratively generates improved solutions to an optimization problem through the marginal changes in previous solutions with the differences in pairs of other previous solutions. The necessary operating parameters used throughout this paper with TDE, were (*ibid*.): amplification control *F* = 0.99, crossover constant *CR* = 0.85, trigonometric mutation probability *M*_*t*_ = 0.05 and number of parameter vectors *NP* = 200. These values are regularly employed in analyses employing TDE (and the original differential evolution), as suggested in Fan *et al*.^15^ For further reference, see Fan and Lampinen^14^ and Storn and Price,^31^ which both include sections describing the sensitivity of the use of different operating parameter values.

When the associations of a number of objects to the states, $d_{ 1} ,d_{ 2} , \ldots ,d_{{n_{\text{D}} }}$, are known, say for the object *o*_*i*_, through the series of association values $a_{i} \left( {d_{ 1} } \right),a_{i} \left( {d_{ 2} } \right), \ldots ,a_{i} (d_{{n_{\text{D}} }} )$, (where $\sum\nolimits_{h = 1}^{n_{\text{D}} } {a_{i} \left( {d_{h} } \right) = 1}$), the effectiveness of a configured NCaRBS system can be measured by a defined objective function (*OB* ^NCaRBS^). Using the well-known Euclidean distance (fit) between each object’s actual $[a_{i} \left( {d_{ 1} } \right),a_{i} \left( {d_{ 2} } \right), \, \ldots ,a_{i} (d_{{n_{\text{D}} }} )]$ vector of association values and pignistic probability derived predicted values’ vector $[BetP_{i} \left( {d_{ 1} } \right),BetP_{i} \left( {d_{ 2} } \right), \ldots ,BetP_{i} (d_{{n_{\text{D}} }} )]$, the *OB* ^NCaRBS^ defined is given as:

$$OB^{\text{NCaRBS}} = \frac{1}{{n_{D} n_{O} }}\sum\limits_{i = 1}^{{n_{o} }} {\sqrt {\left( {BetP_{i} \left( {d_{1} } \right) - a_{i} \left( {d_{1} } \right)} \right)^{2} + \left( {BetP_{i} \left( {d_{2} } \right) - a_{i} \left( {d_{2} } \right)} \right)^{2} + \cdots + \left( {BetP_{i} \left( {d_{{n_{D} }} } \right) - a_{i} \left( {d_{{n_{D} }} } \right)} \right)^{2} } } ,$$

in the limit, since $\sum\nolimits_{h = 1}^{{n_{\text{D}} }} {BetP_{i} \left( {d_{h} } \right) = 1}$ and $\sum\nolimits_{h = 1}^{{n_{D} }} {a_{i} \left( {d_{h} } \right) = 1}$, then 0 ≤ *OB* ^NCaRBS^ ≤ 1. Clearly the intention in any NCaRBS analysis is to minimise the value of *OB* ^NCaRBS^, so as to achieve the best possible model fit between an object’s actual vector of association values and pignistic probability derived predicted values.

## Materials and Methods

In this paper, biomechanical measures from Trendelenburg tests (single-leg stance) were considered for, 13 PA subjects, 14 LA subjects and 16 hips with no pathology (NP), forming a control group.^32^ Quantifying pelvic position during Trendelenburg tests using motion analysis allows subtle differences to be determined for the hip in a static situation,^32^ where significant difference in fontal plane hip moment at 30 s into the test was detected between LA and PA cohorts. During the motion analysis (see Whatling *et al.* 32), each subject was asked to step on to a force plate, to raise and flex the un-supporting leg, holding this position and to returning to the initial position when instructed. In cases of minimal abductor weakness, there may be a delayed positive Trendelenburg test. For this reason, the Trendelenburg test was performed for 1 min on each leg to introduce an element of fatigue into the abductor muscles.

The three Trendelenburg stance characteristics analysed using NCaRBS were C1: pelvic obliquity (angle of unsupported side measured above the horizontal position); C2: frontal plane moment; and C3: frontal plane power acting at the hip, all measured at 30 s into single-leg stance. These measures were selected to identify any changes in frontal plane function (instability and muscle weakness) associated with the surgical approaches, which access the joint either laterally or posteriorly.

The following sections outline the analysis undertaken which is later reported in “Results” section. Firstly the configuration of the NCaRBS model (taken from description of NCaRBS technique in “Background to the NCaRBS technique” section), including the level of model fit achieved when discerning between LA, PA and NP subjects are described. Then the contribution of the considered characteristics to discerning between LA, PA and NP subjects in the configured NCaRBS model is explained. Finally, details of the comparison of this technique with two other commonly used techniques; LDA and NN are given.

### NCaRBS Configuration and Model Fit

From “Background to the NCaRBS technique” section, each subject’s classification to either LA, PA or NP can be represented in vector form, namely when known to be associated with LA (then [1, 0, 0]), PA ([0, 1, 0]) and NP ([0, 0, 1]), with the vector for subject *o*_*i*_ given by [*a*_*i*_(LA), *a*_*i*_(PA), *a*_*i*_(NP)]. Similarly, the predicted association values, in vector form for a subject *o*_*i*_, are represented by [*BetP*_*i*_(LA), *BetP*_*i*_(PA), *BetP*_*i*_(NP)]. It follows, for the THA problem, the objective function *OB* ^NCaRBS^ employed is of the form (described in “Background to the NCaRBS technique” section):

$$OB^{\text{NCaRBS}} = \frac{1}{{3n_{\text{O}} }}\sum\limits_{i = 1}^{{n_{o} }} {\sqrt {\left( {BetP_{i} \left( {\text{LA}} \right) - a_{i} \left( {\text{LA}} \right)} \right)^{2} + \left( {BetP_{i} \left( {\text{PA}} \right) - a_{i} \left( {\text{PA}} \right)} \right)^{2} + \left( {BetP_{i} \left( {\text{NP}} \right) - a_{i} \left( {\text{NP}} \right)} \right)^{2} } } ,$$

used to enable the evaluation of the required control parameters, *k*_*j*,*h*_, *θ*_*j*,*h*_, *A*_*j*,*h*_ and *B*_*j*,*h*_.

Employing the TDE algorithm for constrained optimisation (using its own operating parameters given earlier), with the constraints on the control parameters in NCaRBS (*k*_*j*,*h*_, *θ*_*j*,*h*_, *A*_*j*,*h*_ and *B*_*j*,*h*_), set as; −5 ≤ *k*_*j*,*h*_ ≤ 5, −3 ≤ *θ*_*j*,*h*_ ≤ 3, 0 ≤ *A*_*j*,*h*_ < 1 and 0 ≤ *B*_*j*,*h*_ < 0.6 (based on using a standardised data set, see Beynon),^4^ the objective function *OB* ^NCaRBS^ was optimised (minimised) to the value *OB* ^NCaRBS^ = 0.582 (this was the best *OB* ^NCaRBS^ from five runs undertaken using TDE, see Beynon *et al.* 8 for further discussion of the impact of re-sampling using CaRBS techniques, see also Storn^30^ for brief discussion of ability of DE techniques to attain global optimisation). Using the evaluated control parameters, *k*_*j*,*h*_, *θ*_*j*,*h*_, *A*_*j*,*h*_ and *B*_*j*,*h*_, all the constituent BOEs *m*_*i*,*j*,*h*_(·) can be constructed, and combined to give the necessary object BOEs *m*_*i*_(·), from which the pignistic probability derived predicted values *BetP*_*i*_(LA), *BetP*_*i*_(PA) and *BetP*_*i*_(NP) are found.

With each triplet of pignistic probability derived predicted values *BetP*_*i*_(LA), *BetP*_*i*_(PA) and *BetP*_*i*_(NP) summing to one for each subject, they can be represented as a simplex coordinate in a simplex plot (as in Fig. 1c).

### Contribution of a Characteristic in NCaRBS Model

The contribution described here uses the general constituent BOEs found from the configuration of a NCaRBS system. From the description of the NCaRBS technique, a constituent BOE *m*_*i*,*j*,*h*_(·) associated with a characteristic value *v*_*i*,*j*_ (*i*th subject, *j*th characteristic) contains the mass values offering belief based evidence towards a subject’s association to a state (say {*d*_*h*_}—one of the surgery types LA, PA and NP), its complement ({¬*d*_*h*_}—not *d*_*h*_) and also a level of concomitant ignorance ({*d*_*h*_, ¬*d*_*h*_}—evidence not able to be discerned between *d*_*h*_ and ¬*d*_*h*_).

From the configured NCaRBS system (using the control parameters found), and with the merging of the structural content of parts (a) and (b) in Fig. 1, a series of graphs can be presented graphically showing the contribution contained in the general constituent BOEs formed. That is, for a constituent BOE *m*_*i*,*j*,*h*_(·), the evaluated control parameters *k*_*j*,*h*_, *θ*_*j*,*h*_, *A*_*j*,*h*_ and *B*_*j*,*h*_ define a unique graphical representation of the evidence it contains depending on respective characteristic value (combing the graph parts in Figs. 1a and 1b) which are dependent on the control parameters *k*_*j*,*h*_, *θ*_*j*,*h*_, *A*_*j*,*h*_ and *B*_*j*,*h*_—see later in Figs. 3 and 4).

### Comparison with LDA and NNs

The level of correct classification from NCaRBS was compared with two well-known machine learning multi-class classification techniques, namely LDA and NNs. Comparisons to both LDA and NNs were made with respect to the level of correct classification. The level of fit was also compared between NN (with different numbers of hidden nodes considered) and NCaRBS. It was not possible to compare the contribution of each variable since this is a unique feature to NCaRBS.

## Results

### NCaRBS Configuration and Model Fit

**Figure 2:**
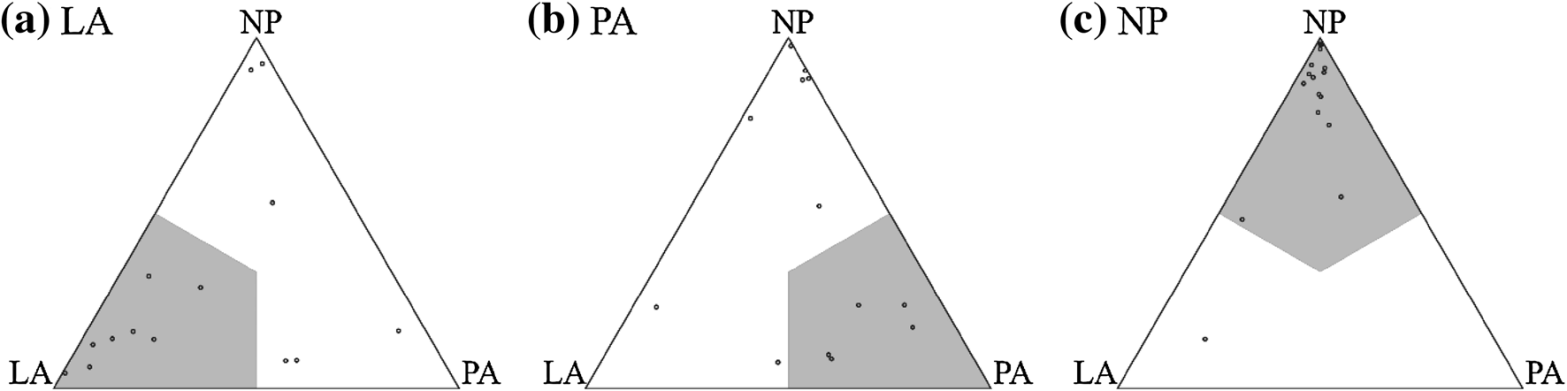
Predicted surgical type associations of subjects by dominant association, who’s actual associations are (a) LA, (b) PA and (c) NP

Figure 2 shows the predicted associations of subjects to the three surgery types, LA, PA and NP, using the triplets of pignistic probability values, in vector form, [*BetP*_*i*_(LA), *BetP*_*i*_(PA), *BetP*_*i*_(NP)]. The three simplex plots presented show subjects’ predicted associations in the form of their respective simplex coordinates, grouped by their known actual surgery type (LA, PA or NP), with the grey shaded regions showing, for an object, where there would be predominance to the correct surgery type (for example a subject known to be associated with surgery type LA would be correctly classified if their prediction value was in the bottom left corner of the simplex plot).

To measure the appropriateness of fit of the predicted association values, the number of simplex coordinates (predicted associations of subjects) that are correctly placed in the appropriate shaded region in the simplex plot in Fig. 2 are: 8 out of 14 LA (57.143%), 5 out of 13 PA (38.462%) and 15 out of 16 NP (93.750%). In total there are 28 out of 43 (65.116%) correctly classified subjects in the concomitant grey shaded regions. This level of fit needs to be taken in context, with only three characteristics describing each subject, features of the considered Trendelenburg test. Further, the intentions of the surgical types, LA and PA, are to give the subjects the possibility of NP, becoming like NP in all respects, so since this analysis is post-surgical the level of fit (classification accuracy) is in regard to how possible it was to still see differences amongst the different subject types.

### Contribution of a Characteristic in NCaRBS Model

Beyond the fit results, the NCaRBS technique is able to offer a series of pertinent graphs elucidating the contribution of the individual characteristics in the predicted associations of the 43 subjects to the three surgery types, LA, PA and NP. Following the previous paragraph, this contribution should be considered in terms of where difference between the surgery types exists, based on the Trendelenburg test, and where these differences are noted across the three subject characteristics (C1, C2 and C3). Two sets of graphs are next described, which offer alternative forms of understanding the contribution of the characteristics.

**Figure 3:**
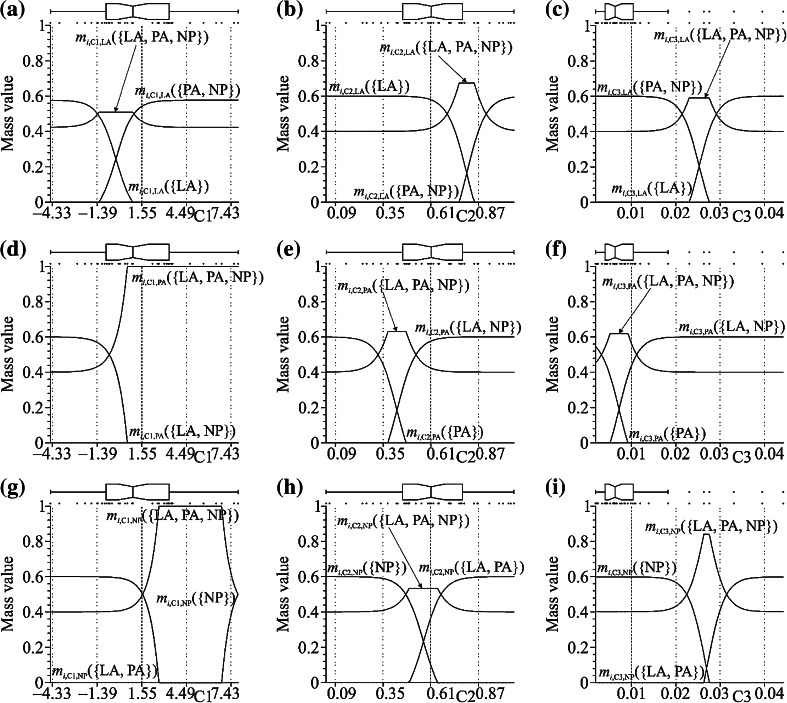
NCaRBS induced graphical representation of the mass values making up the constituent BOEs, *m*_*i*,C?,LA_(·), *m*_*i*,C?,PA_(·) and *m*_*i*,C?,NP_(·), relating to C1 (pelvic obliquity, degrees), C2 (frontal moment, Nm/kg) and C3 (frontal power, W/kg) measured at 30 s into single-leg stance, and the evidence towards the surgery types, LA, PA, and NP and against ({¬LA} = {PA, NP}, {¬PA} = {LA, NP} and {¬NP} = {LA, PA}) and ignorance {LA, PA, NP}

The details presented in Fig. 3 show the contribution contained in the general constituent BOEs and are best understood by initially describing a single graph, in this case Fig. 3a. In Fig. 3a, the constituent BOE *m*_*i*,C1,LA_(·) is described, over the domain bounded by the known C1 (Pelvic Obliquity angle) values in the THA data set, with points and a notched box plot presented at the top to show the specific (points) and statistical (notched box plot) spread of the individual C1 values.

The constituent BOE *m*_*i*,C1,LA_(·) reported in Fig. 3a is made up of three mass values *m*_*i*,C1,LA_({LA}), *m*_*i*,C1,LA_({PA, NP}) and *m*_*i*,C1,LA_({LA, PA, NP}). This constituent BOE is discerning the evidence, from the characteristic value *v*_*i,*C1_ (for C1), on a subject’s association to the surgery type {LA} and not the surgery type {¬LA} = {PA, NP} and a level of concomitant ignorance (assigned to {LA, PA, NP}). The three lines shown in Fig. 3a describe the mass values as the C1 characteristic goes from its identified sample minimum of near −4.457° up to the sample maximum near 7.902°.

In more detail, as the C1 characteristic value increases from its sample minimum of −4.457° up to near −1.235°, there is a gradual decrease in the evidence (mass) associating a subject with surgery type LA and respective increase in the level of concomitant ignorance in the evidence (*m*_*i*,C1,LA_({LA, PA, NP})). From a C1 value near −1.235° up to near 0.942°, the mass value *m*_*i*,C1,LA_({LA}) continues to decrease down to zero, with *m*_*i*,C1,LA_({LA, PA, NP}) remaining constant at 0.510, but an increase (from zero) in the evidence towards a subject being associated with the surgery types PA or NP (*m*_*i*,C1,LA_({PA, NP})). From a C1 value near 0.942° up to its sample maximum of 7.902°, there is a continued increase in the mass value *m*_*i*,C1,LA_({PA, NP}) and decrease in the level of concomitant ignorance *m*_*i*,C1,LA_({LA, PA, NP}) in the evidence. Putting this evidence in summary terms, as the C1 characteristic value increases across the sample domain, there is a gradual ‘non-linear’ shift in the evidence away from a subject having had LA approach to THA towards them having either had the PA approach or NP (with the level of ignorance changing accordingly across this sample domain). Thus suggesting the PA and NP surgical groups demonstrate greater control of their abductors in maintaining a higher angle of the pelvis on the unsporting side. Conversely, the LA surgical group demonstrate greater abductor weakness.^17^

Many of the other constituent BOEs shown in Fig. 3 follow a similar structure, two exceptions to this are the constituent BOEs *m*_*i*,C1,PA_(·) (Fig. 3d) and *m*_*i*,C1,NP_(·) (Fig. 3g). For both these constituent BOEs there are sample sub-domains of C1 (same characteristic in both cases), for where there is only total ignorance in their evidence. That is, for example, in Fig. 3g for *m*_*i*,C1,NP_(·), over the sub-domain 2.677° to 6.806° *m*_*i*,C1,NP_({LA, PA, NP}) = 1.000 (so *m*_*i*,C1,NP_({NP}) = 0.000 and *m*_*i*,C1,NP_({LA, PA}) = 0.000), indicating no evidence associating a subject to either the {NP} or {LA, PA} sets of surgery types.

### Contributions of Subject Characteristics to Surgery Types LA, PA and NP

Individual constituent BOEs, like the BOEs discussed previously, can be combined in a variety of ways to produce further BOEs (see the description of NCaRBS given previously). Here, the three constituent BOEs *m*_*i*,C?,LA_(·), *m*_*i*,C?,PA_(·) and *m*_*i*,C?,NP_(·) can be combined to form a characteristic BOE *m*_*i,*C?,−_(·), which represents all the evidence a particular characteristic offers towards the prediction of a subject’s surgery type. Since for each characteristic, a characteristic BOE *m*_*i,*C?,−_(·) is a BOE made up of seven mass values and focal elements (the power set of {LA, PA, NP} minus the empty set), the pignistic probability function constructs the values *BetP*_*i*,C?_(LA), *BetP*_*i*,C?_(PA) and *BetP*_*i*,C?_(NP) (found from the concomitant characteristic BOE), for a subject, to describe specific levels of associations between the subjects’ characteristics and surgery types LA, PA and NP.

**Figure 4:**
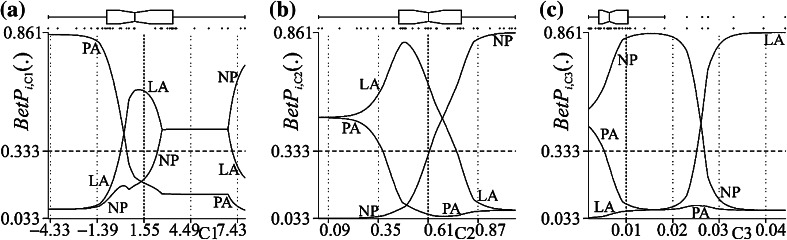
NCaRBS induced graphical representation of the contributions of subject characteristics to surgery types LA, PA and NP, using pignistic probability values, *BetP*_*i*,C?_(LA), *BetP*_*i*,C?_(PA) and *BetP*_*i*,C?_(NP), from characteristic BOEs (a) contribution of C1, (b) contribution of C2, (c) contribution of C3

It follows that sets of constructed *BetP*_*i*,C?_(·) values can be produced from the individual characteristic BOEs, shown over the full domains of the respective characteristics, between the minimum and maximum values present, see Fig. 4.

In Fig. 4, each presented graph shows the movements of the *BetP*_*i*,C?_(LA), *BetP*_*i*,C?_(PA) and *BetP*_*i*,C?_(NP) values, describing levels of evidential association (in terms of pignistic probability) attributed to the surgery types, Lateral Approach (LA), PA and No Pathology (NP), respectively. The graphs presented show these contributions over the range [0.033, 0.861], the bounds found from the least and largest of any *BetP*_*i*,C?_(·) values (instead of the full *BetP*_*i*,C?_(·) range of [0, 1]). As in Fig. 3, point and notched box plots show the specific (points) and statistical (notched box plot) spread of the individual C1 values in the THA data set. These graphs can be compared with the respective constituent BOEs (shown in Fig. 2). The case of C1 is next considered (Fig. 4 *a*).

In Fig. 4a, as the C1 (Pelvic Obliquity angle) characteristic increases from its sample minimum −4.457° to the maximum 7.902°, there is a gradual, but non-linear, decrease in the probable association of a subject to being associated with the PA surgery type. There is a similar gradual non-linear increase over the sample domain of C1 in the probable association of a subject to the NP surgery type. For the surgery type LA, the association is less consistent, over the two sub-domains, −4.457° to 1.198° and 1.198° to 7.902°, there is increasing and decreasing levels of probable association, respectively. It is interesting that for the LA association, the point of change in increasing and decreasing probable association, through inspection of the above notched box plot in Fig. 4a, is very near the median of the sample of C1 characteristic values. Similar inference can be gained from the two other characteristics, C2 (frontal plane hip moment) and C3 (frontal plane hip power), in Figs. 4b and 4c, respectively.

As the C2 characteristic (frontal plane hip moment, Nm/Kg) increases, see Fig. 4b, there is a gradual increase in the probable association of a subject to the NP surgery type. Again for the surgery type LA, the association is less consistent with a firstly increasing (near the median of the sample) then decreasing level of probable association, respectively. There is also a decrease in the probable association of a subject to surgery type PA. This is due to a similar mean hip frontal moment for the LA (0.49 ± 0.22 Nm/Kg) and PA (0.59 ± 0.34 Nm/Kg) surgical types.^32^

As the C3 characteristic (frontal plane power, W/Kg) increases, see Fig. 4c, unlike the previous characteristics (see Figs. 4a and 4b), the domain shown for it is affected by the actual spread of the characteristic values from the population of subjects considered. That is, for the upper half of the shown domain, it is associated with only six values amongst the population (potential outliers), as indicated by the concomitant notched box plot for this characteristic. It follows, the interpretation of its contribution to discern between the three subject types is only considered up to near the midpoint of the presented domain. Over this sub-domain, as the characteristic value increases, there is an increase in the probable association of a subject to the NP surgery type and decrease in probable association to PA surgical.

### Comparison with LDA and NNs

This section briefly describes the results from employing the LDA and NN analysis techniques.

#### Linear Discriminant Analysis (LDA)

Following LDA, 8 out of 14 LA (57.143%), 3 out of 13 PA (23.077%) and 10 out of 16 NP (62.5%) were correctly classified. In total there are 21 out of 43 (48.837%) correctly classified subjects, as indicated in Table 1, which breaks down the actual and predicted classification results.

**Table 1:** Linear discriminant analysis results

Actual	LA	PA	NP
Predicted			
LA	8	4	2
PA	5	3	5
NP	1	5	10

The LDA predicted correct LA classification to the same degree as NCaRBS but is not able to classify NP and PA function as accurately as NCaRBS.

#### Neural Networks

Three models were considered in the NN analyses, when one, two and three hidden nodes were employed in the hidden layer^1^ (with three outputs in the output layer). Table 2 breaks down the actual and predicted classification results for the three cases. An objective function *OB* ^NN,*n*^, (where *n* is the number of hidden nodes) was constructed to mimic the objective function employed with NCaRBS (again denoting 14 LA classified subjects with vector [1, 0, 0], 13 PA as [0, 1, 0] and 16 NP [0, 0, 1]). Moreover, the objective function employed (as with NCaRBS) was based on a Euclidean distance measure of the three outputs from the NN and the three vector formations of subjects classifications, namely one of [1, 0, 0], [0, 1, 0] and [0, 0, 1] in each case.

**Table 2:** Neural network results using 1, 2 and 3 hidden nodes

Actual	One hidden node			Two hidden nodes			Three hidden nodes		
	LA	PA	NP	LA	PA	NP	LA	PA	NP
Predicted									
LA	0	7	7	11	1	2	8	3	3
PA	0	8	5	6	5	2	0	7	6
NP	0	1	15	2	2	12	0	1	15

A NN with one hidden node gave a 53.488% correct classification rate (*OB* ^NN,1^ = 0.658). This is a worse fit and worse correct classification than with the NCaRBS model (*OB* ^NCaRBS^ = 0.582 and correct classification rate of 65.116%).

A NN with two hidden nodes gave a 65.116% correct classification rate. This gives a better fit (*OB* ^NN,2^ = 0.455) and equal correct classification to NCaRBS. This model provides a higher classification rate for LA (21.4% higher than NCaRBS) and PA (7.7% higher than NCaRBS), however the ability to classify NP is inferior to the NCaRBS model.

A NN with three hidden nodes gave a 69.767% correct classification rate. This gives a better fit (*OB* ^NN,3^ = 0.408) and better correct classification to NCaRBS. This model is comparable to NCaRBS in terms of LA and NP classifications and produced a 15.4% higher classification rate for PA subjects.

For clarity and to allow direct comparison, Table 3 summarises the results with the highest classification accuracy from the NCaRBS, LDA and NN approaches.

**Table 3:** Results with the highest classification accuracy from the NCaRBS, LDA and NN approaches

Actual	NCaRBS			LDA			NN (3 hidden nodes)		
	LA	PA	NP	LA	PA	NP	LA	PA	NP
Predicted									
LA	8	3	3	8	4	2	8	3	3
PA	2	5	6	5	3	5	0	7	6
NP	1	0	15	1	5	10	0	1	15
Precision	0.727	0.625	0.625	0.571	0.25	0.589	1	0.636	0.625

## Discussion

This paper has exposited a new technique for *n*-state classification, where objects are associated with a number of different states and described by a series of characteristics. The introduced technique named NCaRBS, is shown to be an important development on the original CaRBS technique (itself only recently introduced^4^,^5^,^7^). As such, the analysis is undertaken through uncertain reasoning, due to the operational rudiments of NCaRBS, like CaRBS, based on the Dempster–Shafer theory of evidence.

Throughout the exposition of NCaRBS, the THA problem has been considered, whereby subjects with and without hip replacement surgeries are investigated based on certain post-operative Trendelenburg characteristics. It follows, the defined three state problem, discerning between subjects association with the surgery types LA, PA or NP, is pertinent to be analysed using NCaRBS.

The results presented include an understanding of the evaluation of fit to a model (configuration of a NCaRBS system), using the pignistic probability function. This fit process is shown to require no assumptions on the independent variables (characteristics) uitlised, as would be necessary in regression based analyses^25^ (indeed it would be non-trivial for a regression based analysis to be performed on this defined three state THA problem). With the model configuration process defined an optimisation problem, here undertaken using Trigonometric Differential Evolution (TDE), future research should investigate the local–global optimisation issue in regard to the impact on NCaRBS results.

This is the first application of NCaRBS to assess outcome of THA where three surgery types, LA, PA and NP were classified using data from static Trendelenburg test. Using only the three characteristics selected, NCaRBS classified NP surgery type with 93.750% accuracy. Therefore this method was able to distinguish between NP and post THA surgery to high accuracy. The functional characteristics used in the classification were able to distinguish the LA with 57.143% accuracy and PA with 38.462% accuracy.

When classifying using the three variables in combination, the PA group had two main clustered groups, patients who exhibited NP function (75% of misclassified subjects being characterised as having NP function) and a group that was clearly different to both LA and NP function. The LA group had a larger variability in the variables selected resulting in weaker classification accuracy and less defined LA characteristic group. 6/8 misclassified PA subjects were classified as having NP function, whereas only 3/6 misclassified LA subjects were. This suggests some differences exist in frontal plane function due to different surgical procedures, and the PA approach restores more NP abductor strength in the frontal plane than the LA. However, further investigation is required using different characteristics. A suggestion for future work is to use principal components of the pelvic obliquity, frontal moment and power waveforms instead of discrete values taken at a specific time point. This would ensure any changes as the subject acts to stabilise their position and due to muscle fatigue will be incorporated in the decision making.

It would also be beneficial to classify hip function during level/stair gait and other daily activities. In a study by Madsen *et al.* 22 of the differences between anterolateral (A–L) and posterolateral approach (P–L), subjects were classified with the following accuracies; 89% for NP, 90% for A–L and 50% for P–L. This high accuracy may be due to the investigation of several functional variables from level gait as opposed to 3 measures from a clinically employed Trendelenburg Test. The subjects in this current analysis were previously classified based on gait variables using a similar classification method^32^ distinguishing between the following groups with the following accuracies: (1) LA and NP = 93.3%; (2) PA and NP = 86.2%; (3) LA and PA = 55.6%. The outcomes from Madsen *et al*.,^22^ Whatling *et al.*,^32^,^33^ and the current study, all imply that greater NP function is restored following PA to surgery.

A comparison was made between NCaRBS, LDA and NN. LDA had lower classification accuracy than NCaRBS, and NN was comparable to NCaRBS when two or three hidden nodes are considered. Using two nodes, classification was improved for LA and PA but was worse for NP. Using three nodes, the classification of PA was improved compared to NCaRBS. Clearly, as more hidden nodes are considered with NN, the model fit and subsequent correct classification rate improved. It was observed that the NN classification results were crisp (with certainty), with 0 s and 1 s appearing the relative prediction vectors for subjects, unlike with NCaRBS where there was ambigiuous classification (points inside the simplex plot not at a vertex). This is a reason as to why for NN with two hidden nodes the level of fit is better than with NCaRBS, but the same classification accuracy. This would be mean, for incorrect classified cases, where with NCaRBS a case’s position in the simplex plot would help see the ambiguity of their incorrect classification, with NNs the incorrect classification would be with 100% certainty even though that certainty is to the wrong classification. This is an important point when considering biomechanical data, which can be conflicting or corroborating in nature. A range of functions or outcomes is to be expected for human subjects. They will not all function the same if they belong in the same group and therefore being able to see the ambiguity in classification is a necessary and important facet, inherent with the NCaRBS technique. A technique with the ability to quantify and visualise the ambiguity in a classification, along with the contributions of each variable to the classification, is highly valuable. It provides useful feedback on cohorts and individual subjects, facilitating clinical interpretation of the results. Such tools, with further development, may be useful in clinical and rehabilitation settings. Wickramarathne *et al*.^34^ describes measures that facilitate the comparison of crisp (with certainty) and more ambiguous predictions. We report precision for each classification in Table 3, allowing direct comparison of the results. The use of other performance measures mentioned in this paper are beyond this remit of the current study, but do offer a potential way forward in future comparison focused studies.

A noticeable positive of the employment of the NCaRBS technique is the level of elucidation able to be undertaken on the contribution of the characteristics (independent variables) used to describe the objects (subjects) and their associations to the three states (surgery types LA, PA, NP). This is in part due to the homogeneity of the role played by the DST based bodies of evidence (BOEs), in quantifying the evidence affected by the characteristic, and in particular the method of combination of BOEs.

Indeed, the contribution results introduce a novel way of viewing the combination of characteristics, namely (as shown in Fig. 3), the ability to see how, as a characteristic values changes, the evidence towards the association of a subject to a state (*d*_*h*_) and not-the-state (¬*d*_*h*_) changes relatively, including also the changes in the concomitant ignorance with the characteristic based evidence. Further, through again the use of the pignistic probability function (shown in Fig. 4), the contribution of a characteristic across considered states can be investigated simultaneously.

The benefit of using the current method is the ability to classify function into three characteristic groups and produce simplex plots for visualisation. The contribution diagrams provide a visual explanation of the influence of each characteristic in the classification. This is useful when examining how the trends of each characteristic relate to each hip condition. It is important to be able to not only detect differences between groups but to understand what is contributing to this decision. Other existing algorithms are not able to explain this type of multivariate analysis and contributions in the same way or visually.

The future pertinence of the applicability of the NCaRBS technique will come from the increased familiarity of it operations, and the way it is able to present its findings, using the simplex plot approach to data representation for example. There is an important future positive of the employment of the NCaRBS technique, not needed in this study, namely that the original CaRBS technique^4^ is able to analyse incomplete data, without the need for the management of the missing values present. This could potentially make NCaRBS a most feasible technique when dealing with patient datasets, and certainly something to consider in the future. This technique is versatile and can be used for a diverse range of datasets (including different sized data sets, see CaRBS related papers). The current study however, has not explored the scalability of this technique and this needs to be formally assessed with larger datasets.

This study introduces a new application of NCaRBS to the biomechanics field to characterise LA, PA and NP function. From this investigation of the Trendelenburg test, it appears that there are three distinct functional groups relating to NP, PA and LA, however there is also an element of overlap in function resulting in misclassified subjects. The power of NCaRBS has been demonstrated on this simple analysis of three variables from a Trendelenburg test and will be used in future studies with larger datasets. It has also been compared favourably to LDA and NN techniques.
